# High-Affinity Peptides for Target Protein Screened in Ultralarge Virtual Libraries

**DOI:** 10.1021/acscentsci.4c01385

**Published:** 2024-11-02

**Authors:** Boyuan Xue, Ruixue Li, Zhao Cheng, Xiaohong Zhou

**Affiliations:** † Center for Sensor Technology of Environment and Health, School of Environment, Tsinghua University, Beijing 100084, China

## Abstract

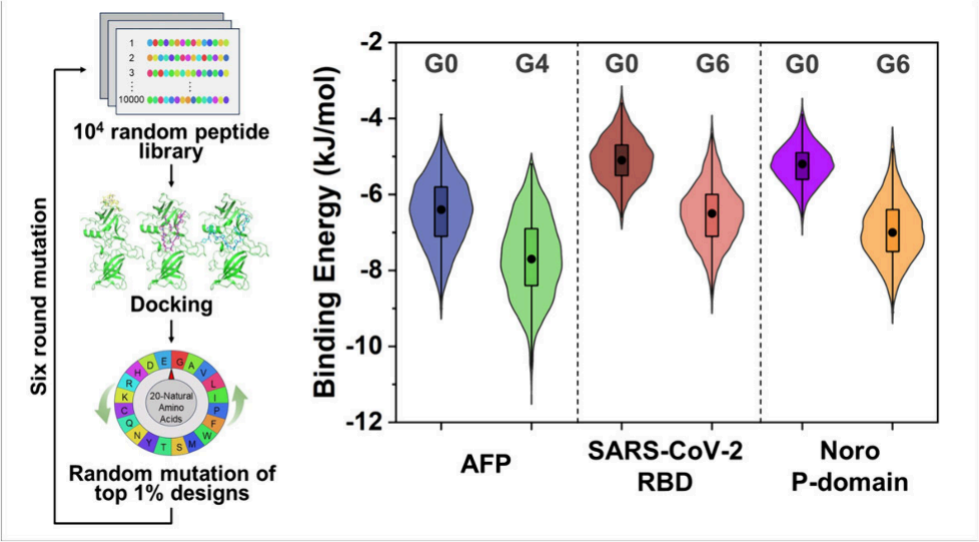

High-throughput virtual screening (HTVS) has emerged as a pivotal strategy for identifying high-affinity peptides targeting functional proteins, which are crucial for diagnostic and therapeutic applications. In the HTVS of peptides, expanding the library capacity to enhance peptide sequence diversity, thereby screening out excellent affinity peptide candidates, remains a significant challenge. This study presents a *de novo* design strategy that leverages directed mutation driven HTVS to evolve vast virtual libraries and screen peptides with ultrahigh affinities for various target proteins. Utilizing a computer-generated library of 10^4^ random 15-mer peptide scaffolds, we employed a self-developed algorithm for parallelized HTVS with Autodock Vina. The top 1% of designs underwent random mutations at a rate of 20% for six generations, theoretically expanding the library to 10^14^ members. This approach was applied to various protein targets, including a tumor marker (alpha fetoprotein, AFP) and virus surface proteins (SARS-CoV-2 RBD and norovirus P-domain). Starting from the same 10^4^ random 15-mer peptide library, peptides with high affinities in the nanomolar range for three protein targets were successfully identified. The energy-saving and high-efficient design strategy presents new opportunities for the cost-effective development of more effective high-affinity peptides for various environmental and health applications.

## Abstract 2

This work devised a *de novo* design strategy that leverages directed mutation driven HTVS to evolve vast virtual libraries and screen peptides with ultrahigh affinities for various target proteins.

## Introduction

Rapidly developing hyper-stable inhibitors that bind specifically and sensitively to functional proteins is beneficial for diagnostics and therapeutics.^1,2^ Among them, small peptides usually with fewer than 50 amino acids in sequence have been widely exploited due to their merits over other biomaterials.^3^ Small peptides can be chemically synthesized, hence allowing for large-scale production at lower manufacturing costs, while also eliminating the potential contamination of cellular materials.^4,5^ Peptides can be readily modified or functionalized at specific sites, enabling their conjugation with nanomaterials, imaging probes, or other materials for diagnostics and therapeutics.^6^ Furthermore, small peptides exhibit weak immunogenicity, possess good tissue (tumor) penetration, and when appropriately modified with cell-penetrating agents, they can directly traverse the cell membrane and localize within the cytoplasm to exert therapeutic effects.^7^ By leveraging the aforementioned excellent properties, peptides demonstrate wide-ranging applications in fields such as drug therapy, imaging probes, affinity purification, and detection sensing.^1^

Computer-based high-throughput virtual screening (HTVS) technology has been proven to be a powerful and efficient way to screen affinity candidates from various biomolecular libraries.^8−10^ This technology ranked the complementarity of target proteins and biomolecules in the library at the atomic level and further predicted their interaction affinities, thereby screening the potential high-affinity biomolecules for targets. In HTVS, docking programs such as DOCK, GOLD, FlexX, GLIDE, AutoDock, MOE-Dock, and Surflex-Dock, as well as network-based servers like PatchDock, HEX, and HADDOCK, are widely used for high-throughput docking analysis of biomaterials and their target proteins.^1,11^

Generating large-scale peptide libraries is a crucial step in HTVS. In peptide library construction, one of the main approaches involves creating libraries based on the resolved crystal structure of the affinity protein binding for the receptor (i.e., target protein), while the other is generating random peptide libraries.^12^ Between both of them, random libraries, randomly generated peptides of a set of length, or a range of lengths, are expected to identify novel binding partners of a target of interest. Taking severe acute respiratory syndrome coronavirus 2 (SARS-CoV-2) as an example, all lineages of SARS-CoV-2 share the viral entry through the spike protein receptor-binding domain (RBD) interaction with human angiotensin-converting enzyme 2 (hACE2). Therefore, the design of peptides derived from the structure of hACE2 for RBD binding by extracting the partial helices and folds from hACE2, reconstructing the scaffolds based on the hotspot residues of resolved RBD-hACE2 binding surface,^13−16^ or even generating randomly scratches without relying on known RBD-binding interactions^17,18^ has been widely explored. The second approach starts its design completely from scratch, without relying on known protein-binding interactions, which makes it highly scalable. In this approach, the range of possibilities for design is much larger, and so potentially, a greater diversity of high-affinity peptide binding modes can be identified. Due to limitations in computing power, currently generated random peptide libraries typically consist of ultrashort peptide sequences containing three to four amino acids, forming 10^3^ to 10^4^ members.^19−23^ Hence, expanding the library capacity to enhance peptide sequence diversity, thereby screening out excellent affinity peptide candidates, remains a significant challenge.

Here, we devised a *de novo* design strategy to design high-affinity peptides for target proteins. A computer-generated library with 10^4^ random peptide scaffolds was built and docked with the target protein via a self-developed algorithm for parallelized HTVS by using Autodock Vina. The top 1% designs in the rankings underwent random mutations with a mutation rate of 20% to form a new peptide library for the next round of docking. After six generations of mutations, the peptide library capacity was theoretically expanded to 10^14^ members, of which the peptides with the strongest binding affinity were selected. As proof-of-concept demonstrations, this strategy is employed to design peptides for a variety of target proteins, ranging from a tumor marker (alpha fetoprotein, AFP) to two virus surface proteins (SARS-CoV-2 RBD and norovirus P-domain), with ultrahigh affinities in the nanomolar range. The hyperstable *in silico* designed high-affinity peptides are easy-to-synthesize and cost-effective, providing a starting point for early diagnostics and therapeutics.

## Results and Discussion

### *De Novo* Design Strategy

**Figure 1:**
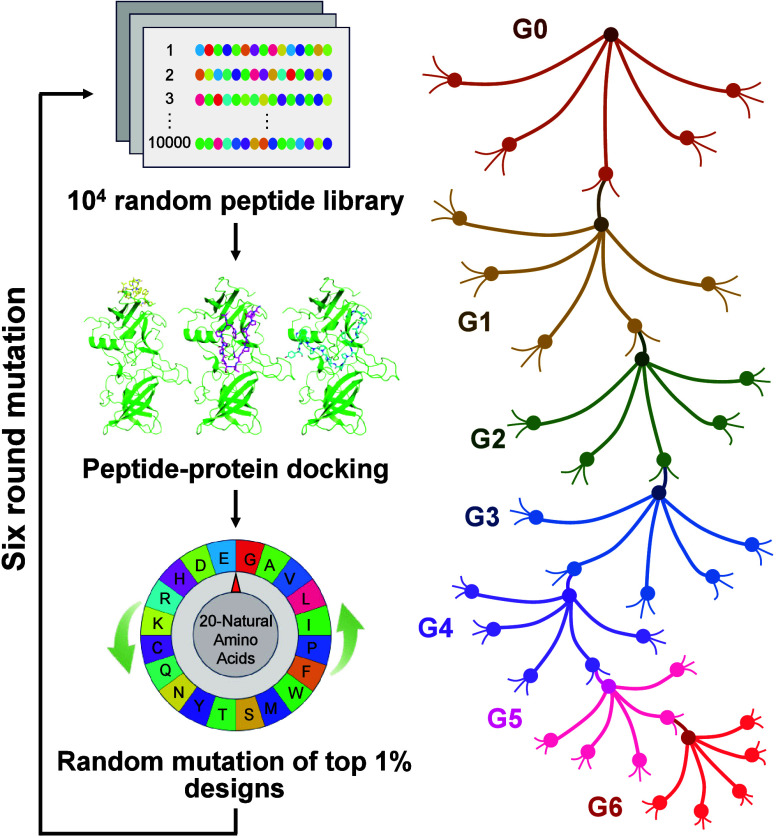
Schematic illustration of directed mutation driven HTVS approach to screen high-affinity peptides for the target protein by mimicking the natural evolution (G0, the original random peptide library; G1–G6, mutant peptide libraries from the first to the sixth generation).

Although it is a common way to construct peptide libraries based on the existing crystal structure of the affinity protein binding for the target, or searching for similar reference proteins in the structure database as a starting point for the design, it has a very limited scope for exploring unknown binding modes.^24^ To expand the library’s capacity and enhance peptide sequence diversity, thereby identifying excellent affinity peptide candidates with potentially unknown binding modes, we proposed the directed mutation driven HTVS approach to screen high-affinity peptides for target protein by mimicking the natural evolution (Figure 1).

In this approach, 10^4^ random 15-mer peptide scaffolds (G0) were computer-generated and modeled from a 1D sequence to a 3D structure. Then, the 3D modeled peptide library and the target protein were used as the input of the system, and the molecular docking and scoring of Autodock Vina were performed by the supercomputer platform, and peptides with high affinity to the receptor were virtually screened out. Using the top 1% peptide designs as the parent, mutants were artificially created by introducing random point mutations into the peptide sequences. The mutants were integrated into a next-generation peptide library for the next round of docking. These steps were repeated for *n* generations until the best designs were obtained. In this study, the mutation generation was set to 6, the mutation rate was 20%, the peptide number was 10 000 per generation, the screening rate was 1%, and the corresponding mutation number was 100 per peptide. After six generations of such evolution (G1–G6), the theoretical peptide library capacity was expanded to 10^14^ members, of which three peptides with the strongest binding affinities were selected.

Timing is critical in a pandemic outbreak; potent diagnostics and therapeutics are needed in as short a time as possible. Designing high-affinity peptides from scratch using random libraries requires immense computing power. To address this challenge, the directed mutation driven HTVS approach provides virtual peptide libraries with a capacity of up to 10^14^ peptide chains, enabling the discovery of high-affinity peptides for the target protein in a computationally affordable manner. Moreover, this approach is versatile and universal; we can use the same 10^4^ peptide library as a starting point to screen for high-affinity peptides targeting different proteins.

### Enhanced Parallelized HTVS Using Autodock Vina

The protein–peptide virtual screening process is a highly complex task which involves high-throughput optimization of peptide libraries, sequential docking, efficient file handling, scoring, parsing, and consolidation of docking results.^25^ AutoDock is popular and widely used software for protein–ligand docking. It commits only to a single CPU per docking run.^26^ AutoDock Vina is the improved version of AutoDock that uses a gradient optimization process for scoring the binding affinity of the ligands. It also features multithreading capability and higher accurate prediction of the ligand binding energy, thus making it a preferred tool for multiple ligand screening processes.^27^ In our study, this limitation was addressed by harnessing the modular programming capabilities of Python to integrate diverse functionalities from PeptideBuilder and MGLTools. Through the development of a bespoke script, the conversion of peptide sequences was automated into pdbqt structural files, thereby circumventing the requirement of Autodock Vina for predefined molecular structure inputs during peptide–protein batch molecular docking procedures. This innovation enables the fully scripted execution of virtual screening for peptide ligands at a scale of 1 × 10^4^, utilizing textual sequence inputs and receptor protein structures as the sole prerequisites.

**Figure 2:**
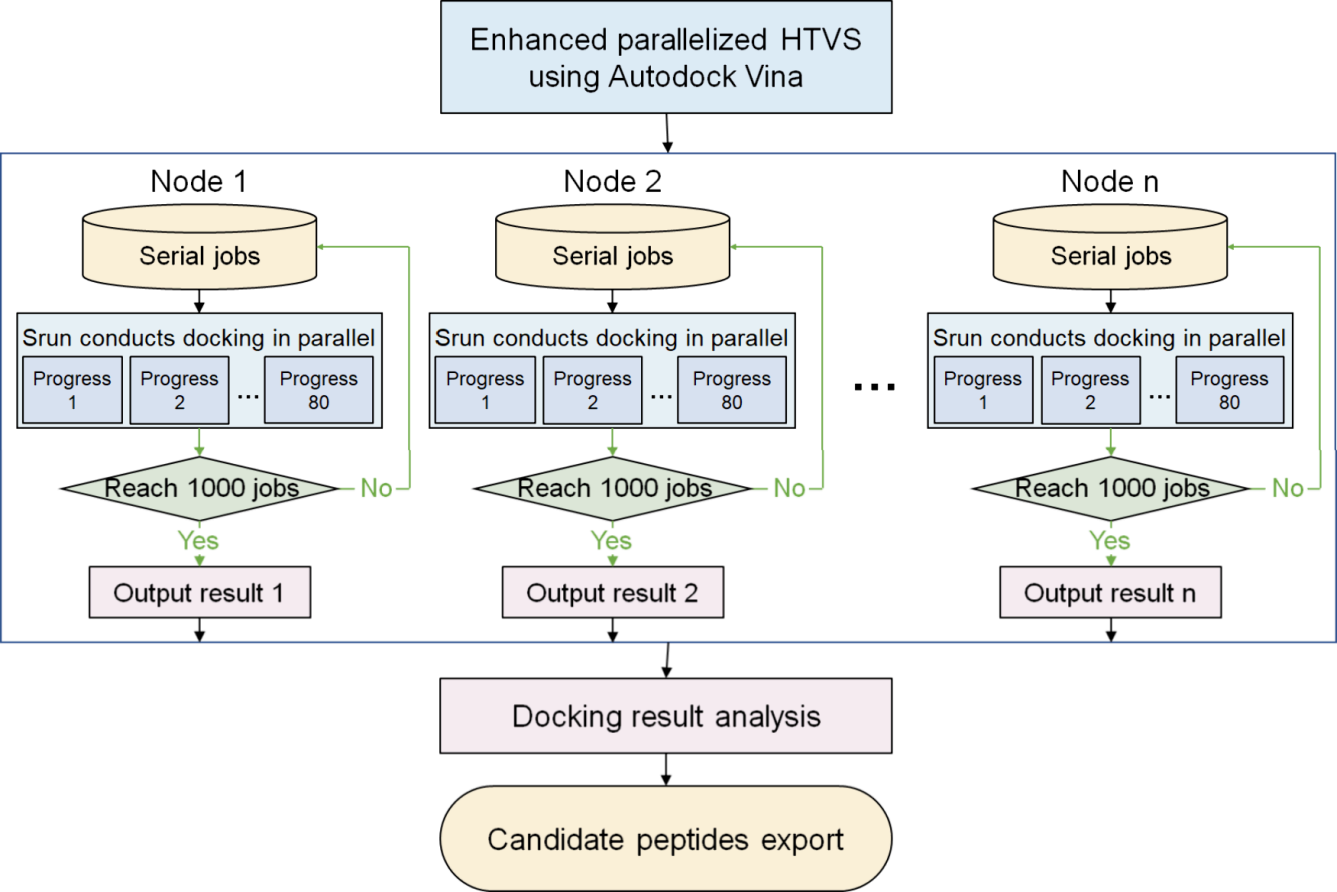
Schematic illustration of a self-developed algorithm for enhanced parallelized HTVS by using Autodock Vina.

To ensure both the expeditious execution and cost-effectiveness of HTVS, our research adopted commercial cloud computing clusters as the underlying hardware infrastructure. We tailored our scripts to align with the SLURM job management system of the cluster, which governs job scheduling and resource allocation on the cluster. This alignment led to the establishment of an enhanced parallelized HTVS approach using Autodock Vina (Figure 2). In contrast to the traditional sequential virtual screening workflow (Figure S1), where tasks are processed one after another, this novel approach enabled the simultaneous activation of numerous computational nodes. Through orchestration, the docking operations were effectively distributed among these nodes. Each node conducted 80 AutoDock Vina jobs in parallel with an exhaustiveness of 1, running as single segmented 4 GB/core CPU threads per job. By adoption of this enhanced parallelized HTVS, there was a notable escalation in screening velocity and an improvement in the robustness of the computational pipeline. Consequently, the screening of peptide libraries on the order of 10^4^ could be achieved within mere hours, showcasing the power and efficiency of this parallelized strategy. This not only optimized resource utilization but also led to a more stable and scalable computational environment for conducting large-scale virtual screening projects.

### Case Study

To test the ability of the directed mutation driven HTVS approach to screen high-affinity peptides for different target proteins, we demonstrated the design of a variety of protein targets, ranging from a tumor marker (alpha fetoprotein, AFP) to two virus surface proteins (SARS-CoV-2 RBD and norovirus P-domain). Three-dimensional crystal structures of three target proteins were obtained from the RCSB Protein Data Bank (http://www.rcsb.org) for design. Human AFP is a tumor-associated fetal mammalian glycoprotein involved in ontogenic and oncogenic growth.^28^ This tumor marker, 70-kDa single polypeptide chain containing 3% to 5% carbohydrate, is encoded by the AFP gene on chromosome 4q25.^29^ ID 7YIM, i.e., cryo-EM structure of human AFP, was used for design.^30^ The COVID-19 pandemic caused by SARS-CoV-2 infection is an ongoing global health threat. All lineages of SARS-CoV-2 share the viral entry through the spike protein receptor-binding domain (RBD) interaction with hACE2 and then trigger the SARS-CoV-2 infection process. The spike protein RBD structure of SARS-CoV-2 was obtained from ID 6M17, i.e., the 2019-nCoV RBD in complex with ACE2-B0AT1.^18^ Norovirus is the leading cause of vomiting and diarrhea and foodborne illness in the United States and other countries.^31^ The P-domain structure of the major capsid protein of Norovirus GII.4 was obtained from ID 7JIE, i.e., the GII.4-Sydney P-domain in complex with the NORO-320 monoclonal antibody, for design.^32^

**Figure 3:**
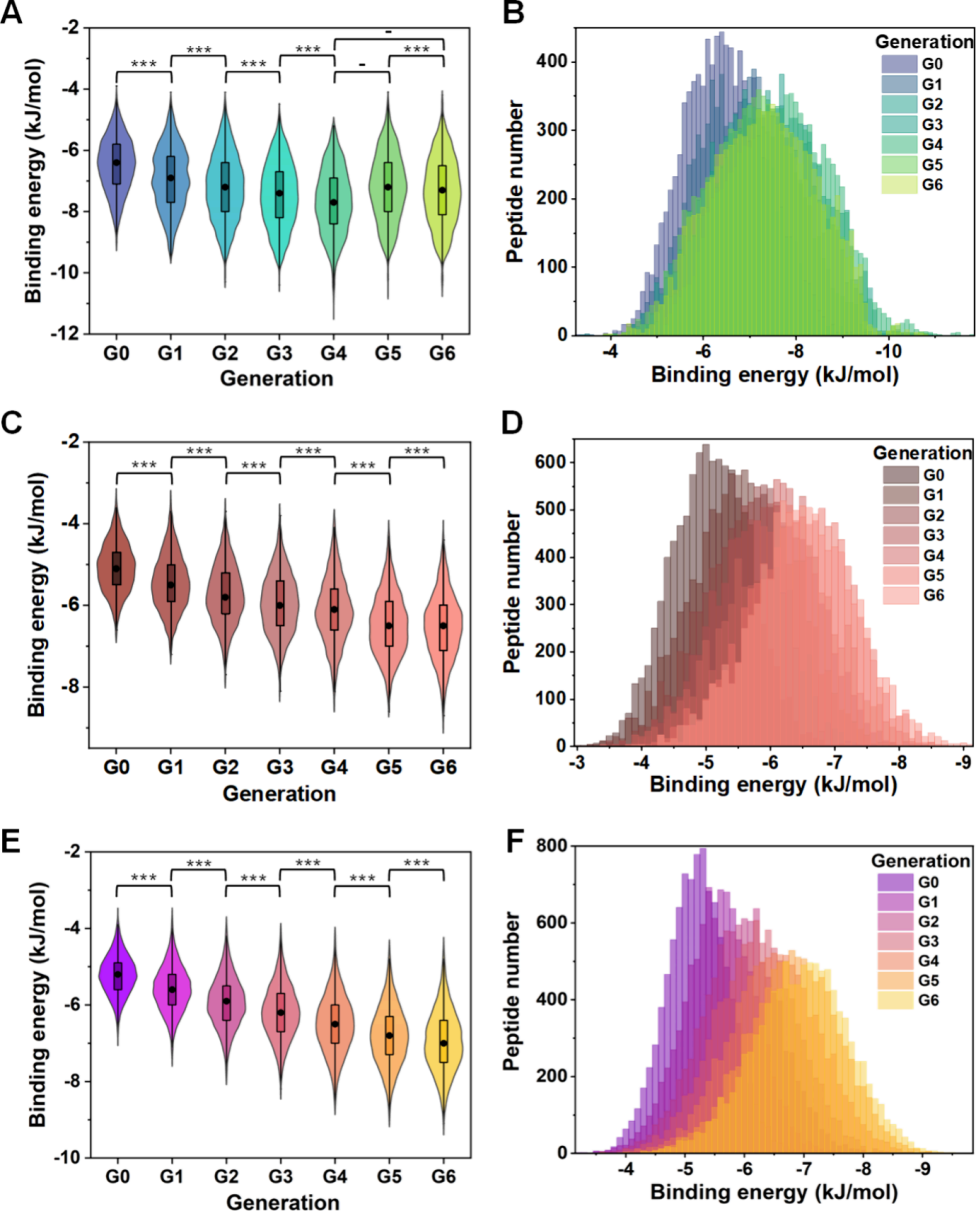
Distribution of binding energies of 10^4^ peptide–target protein complex in different generations of evolution. (A, C, E) Violin plots and Student’s *t* test results comparing binding energy of peptides binding with (A) AFP, (C) RBD of SARS-CoV-2, and (E) Noro P-domain in different mutant generations. Each violin plot includes a boxplot showing the median (black dot), interquartile range (box), and overall range with outliers removed (95% confidence interval, vertical black line). The colored regions provide a kernel density estimation representing the distribution of the data. For the Student’s *t*-test between groups of 10^4^ samples (one-tailed test: whether the binding energy of the group shows a significant decrease over its predecessor), significant differences are represented with asterisks (-, *p* ≥ 0.05; *, *p* < 0.05; **, *p* < 0.01; ***, *p* < 0.001; details in **Table S1**). (B, D, F) Overlapped distribution of binding energies of peptides and (B) AFP, (D) RBD of SARS-CoV-2, and (F) Noro P-domain in different mutant generations.

Starting from the same 10^4^ random 15-mer peptide library, we compared the binding energies of the 10^4^ peptide–target protein complex in different generations of evolution (G0–G6, Figure 3). Utilizing *t*-tests and violin plots, we observed a consistent trend of decreasing binding energy across generations for both SARS-CoV-2 RBD and Noro P-domain (Table S1 and Figure 3C,E), aligning with the principles of directed evolution, wherein successive generations of affinity peptides are engineered to exhibit progressively stronger affinities toward their target molecules. For AFP (Table S1 and Figure 3A), the binding energies of G0–G4 demonstrated a significant increase over their predecessors. However, G5 did not exhibit a statistically significant decrease over G4, and although the G6 showed a significant elevation over G5, it failed to outweigh the G4 significantly. These findings suggested that G4 of AFP reached an optimized level of binding energy. Considering that our selection process prioritized peptides within the top 1% of binding energies, we selected the generation with the lowest binding energy as the best generation (G4 for AFP and G6 for SARS-CoV-2 RBD and Noro P-domain) to proceeded with further validation. For AFP (G4), the average binding energy was measured at −7.668 kJ/mol, with a standard deviation of 1.083 kJ/mol, spanning a score range from −11.8 to −3.4 kJ/mol (Figure 3B). For SARS-CoV-2 RBD (G6), the average binding energy was recorded at −6.518 kJ/mol, with a standard deviation of 0.797 kJ/mol, covering a score range from −9.1 to −3.9 kJ/mol (Figure 3D). Last, for the Noro P-domain (G6), the average binding energy was determined to be −4.930 kJ/mol, with a standard deviation of 0.502 kJ/mol, encompassing a score range of −6.6 to −3.1 kJ/mol (Figure 3F).

**Figure 4:**
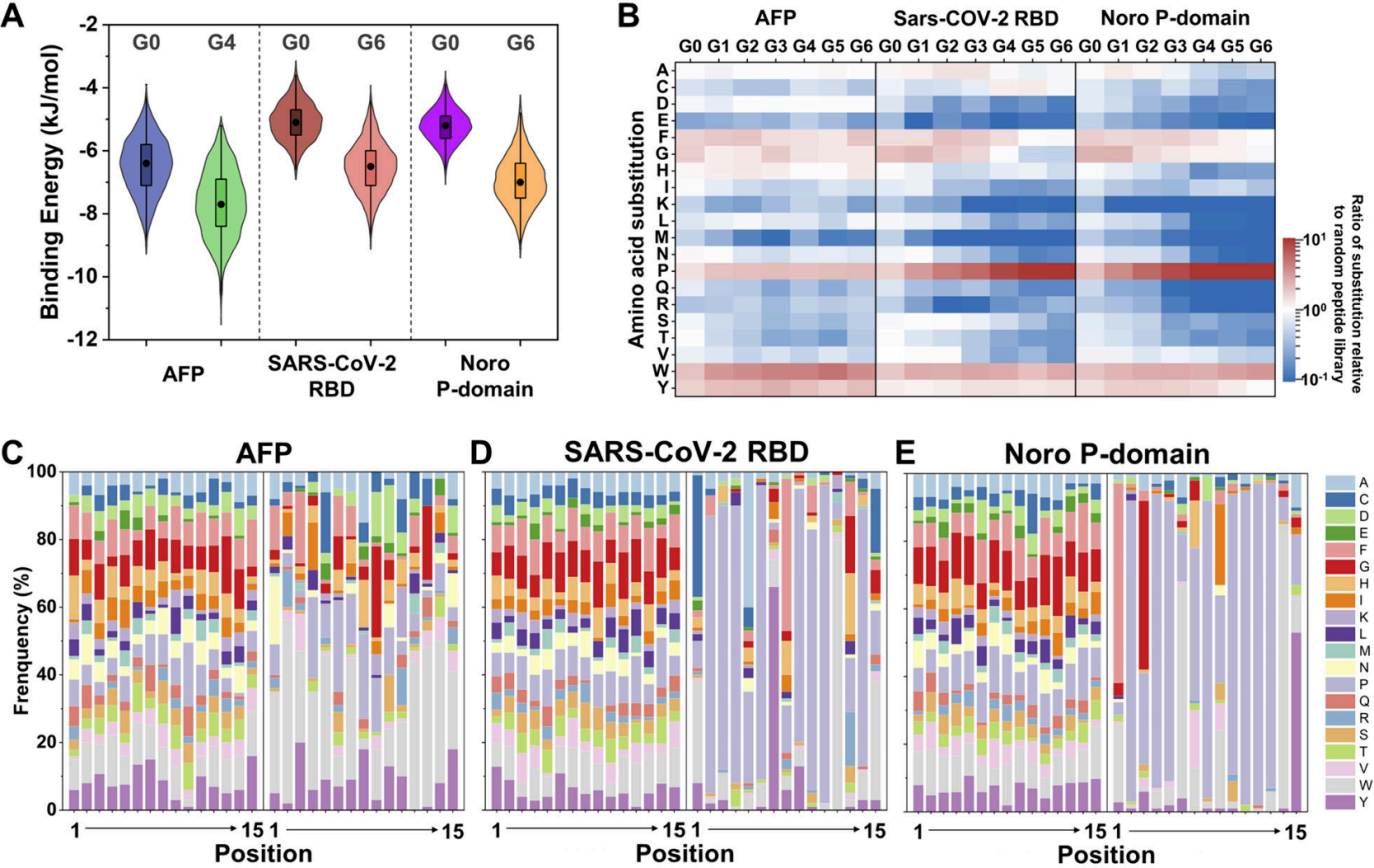
In-depth analysis of the mutant peptide libraries of directed mutation driven HTVS. (A) Violin plots of the energy gap of peptides binding with AFP, RBD of SARS-CoV-2, and Noro P-domain between G0 and the best generation. Each violin plot includes a boxplot showing the median (black dot), interquartile range (box), and overall range with outliers removed (95% confidence interval, vertical black line). The colored regions provide a kernel density estimation representing the distribution of the data. (B) Heat map comparing the proportion of different amino acids of the top 1% peptides in different mutant generations (ratio to the amino acid proportion of original random peptide library). (C–E) Amino acid composition at different positions of peptide sequences with the top 1% peptides binding with (C) AFP, (D) RBD of SARS-CoV-2, and (E) Noro P-domain for G0 and the best generation with the lowest binding energy.

Notably, compared to the original random peptide library, after six generations of iterative mutation, the lowest binding energy between the peptide libraries and target proteins achieved a significant reduction of 1.7 to 2.3 kJ/mol, fully demonstrating the superior performance of our approach (Figure 4A). Furthermore, we conducted a meticulous analysis of the amino acid composition of the top 1% of peptides for each generation (Figure 4B). It revealed the significant changes in the proportion of different amino acids during the mutation process for peptides targeting SARS-CoV-2 RBD and Noro P-domain, particularly proline (P), which surged from less than 10% to approximately 50%. In contrast, the proportion of various amino acids in peptides targeting AFP did not undergo drastic changes, with their evolutionary features more reflected in the differences in amino acid order. Further, we delved into the amino acid composition at different positions of peptide sequences with top 1% peptides for G0 and the best generation. For AFP, this analysis further confirmed that the optimized peptide library underwent significant changes in amino acid order rather than composition (Figure 4C). In contrast, the peptide libraries binding with SARS-CoV-2 RBD and Noro P-domain experienced significant changes in amino acid composition, which manifested in the specialization of amino acid proportion at specific positions (Figure 4D,E).

For the top 0.3% peptides with the highest affinity, i.e., 30 peptides for AFP with scores less than −10.6 kJ/mol, 30 peptides for the SARS-CoV-2 RBD with scores less than −8.7 kJ/mol, and 30 peptides for the Noro P-domain with scores less than −9.0 kJ/mol, we extracted their sequences and calculated their physical and chemical properties (Tables S2–S4, top 10 exemplified in Table 1). The molecular weights of the peptides for AFP (Table S2), SARS-CoV-2 RBD (Table S3), and the Noro P-domain (Table S4) ranged from 1808 to 2249, 1626 to 2113, and 1617 to 2029 Da, respectively. In terms of hydrophobicity, with the exception of three peptides for AFP that exhibited negative values, all other peptides possessed positive values, indicating that peptides with a certain degree of hydrophobicity exhibit a stronger affinity for the three targets. It is worth noting that for AFP, the acquisition of three hydrophilic peptides may be caused by different interaction regions with the target. This observation indirectly underscores the advantage of directed mutation driven HTVS, which can circumvent the risk of becoming trapped in local minima, thereby enabling the selection of peptides with higher affinity from a broader range. Regarding net charge, there were both positively and negatively charged peptides under neutral pH conditions, suggesting that our HTVS method can identify strong affinity peptides across a wider spectrum of possibilities.

**Table 1:** Sequences and Main Physical and Chemical Properties of the Top 10 Peptides Binding with the Three Targets

target	no.	sequence	molecular weight (Da)	hydrophobicity	net charge (pH 7)	docking score (kJ/mol)
AFP	7937	WWWWWPHHDWKLVWC	1970	0.50	–2.05	–11.8
	1775	NWFPCFWAIDDWWIA	1927	0.32	–2.05	–11.5
	474	AWYDGYFGPDCWWNF	1989	0.18	–0.54	–11.5
	5890	PIWWYHAEKPEHWPI	1912	0.23	1.22	–11.5
	4687	GWFRGQPYFGHFWLN	1983	0.39	–0.02	–11.5
	3816	WLIFDTPGPFYWRWV	2017	0.33	–0.78	–11.4
	6973	WWALEHYPPPYMQIW	1974	0.11	1.45	–11.4
	5856	MSWWYHAGKPQHWPY	1917	0.39	–1.08	–11.4
	1751	NNFPCFWCGDWWGWV	1909	0.34	1.45	–11.1
	6002	PIFWVHAYIPRHLPY	2249	0.33	0.22	–11
SARS-CoV-2 RBD	1298	YPPPAPYSPPPWIPQ	1764	0.33	–0.05	–9.1
	2458	CPPPAPQFPFPWPPW	1741	0.44	–0.05	–9.1
	3286	WPPPCPIFPPPLPPW	1689	0.17	0.95	–9.1
	1927	CPPPFPGRPPPWPPF	1849	0.22	0.46	–9
	9913	WPPPPPYFPPPWHHP	1762	0.01	–0.05	–9
	6985	CPRPPPYDPPPWGPW	1835	0.38	–0.09	–9
	9811	WFPPPPYFPPCWTCP	1885	0.18	0.42	–9
	393	CPYPNPAHWPPWHPW	1644	0.03	1.18	–9
	6626	CPPPPPYYKPPGHPP	1944	0.19	0.98	–9
	7068	PPPPWTWFPPPWRPW	1760	0.43	–0.05	–9
	1107	WPAPYAPPVPPPPWY	1717	0.28	0.22	–9.8
Noro P-domain	21	FPPPPPHPWPPPPWA	1779	0.39	–0.02	–9.5
	3076	FFGPPPWPPPPPPWY	1744	0.40	–0.02	–9.5
	2500	WPGPPWGPIPPPPWY	1887	0.44	–0.03	–9.4
	5238	FPYPPWVPPFPPWPY	2029	0.39	–1.02	–9.4
	4305	WDWPPWVPWPPPPWW	1988	0.35	–1.02	–9.3
	595	FPPPPWWDWPPPPWW	1810	0.33	–0.02	–9.3
	7725	WPYPPWVPPPPPWPP	1768	0.44	–0.02	–9.3
	1368	FPGPWWPPIPPPPWP	1745	0.31	0.22	–9.2
	29	FPPPPPHPWPPPPWV	1736	0.30	0.22	–9.2

We further utilized PepATTRACT to conduct small-scale molecular docking analysis of the top 0.3% peptide sequences with target proteins (Figure S2A). Compared with PepATTRACT scoring results of G0 (Figure S2B), our strategy significantly reduced the affinity scores, with an average decrease ranging from 1 to 5 kcal/mol depending on the targets, thereby validating the efficacy of directed mutation driven HTVS to screen peptides with higher affinity. By integrating the scoring results from Autodock Vina and PepATTRACT, the top three designs were selected for subsequent BLI analysis: Pep-5890, Pep-6973, and Pep-7937 for AFP; Pep-1927, Pep-2458, and Pep-6626 for Sars-CoV-2 RBD; and Pep-21, Pep-595, and Pep-3076 for the Noro P-domain.

**Figure 5:**
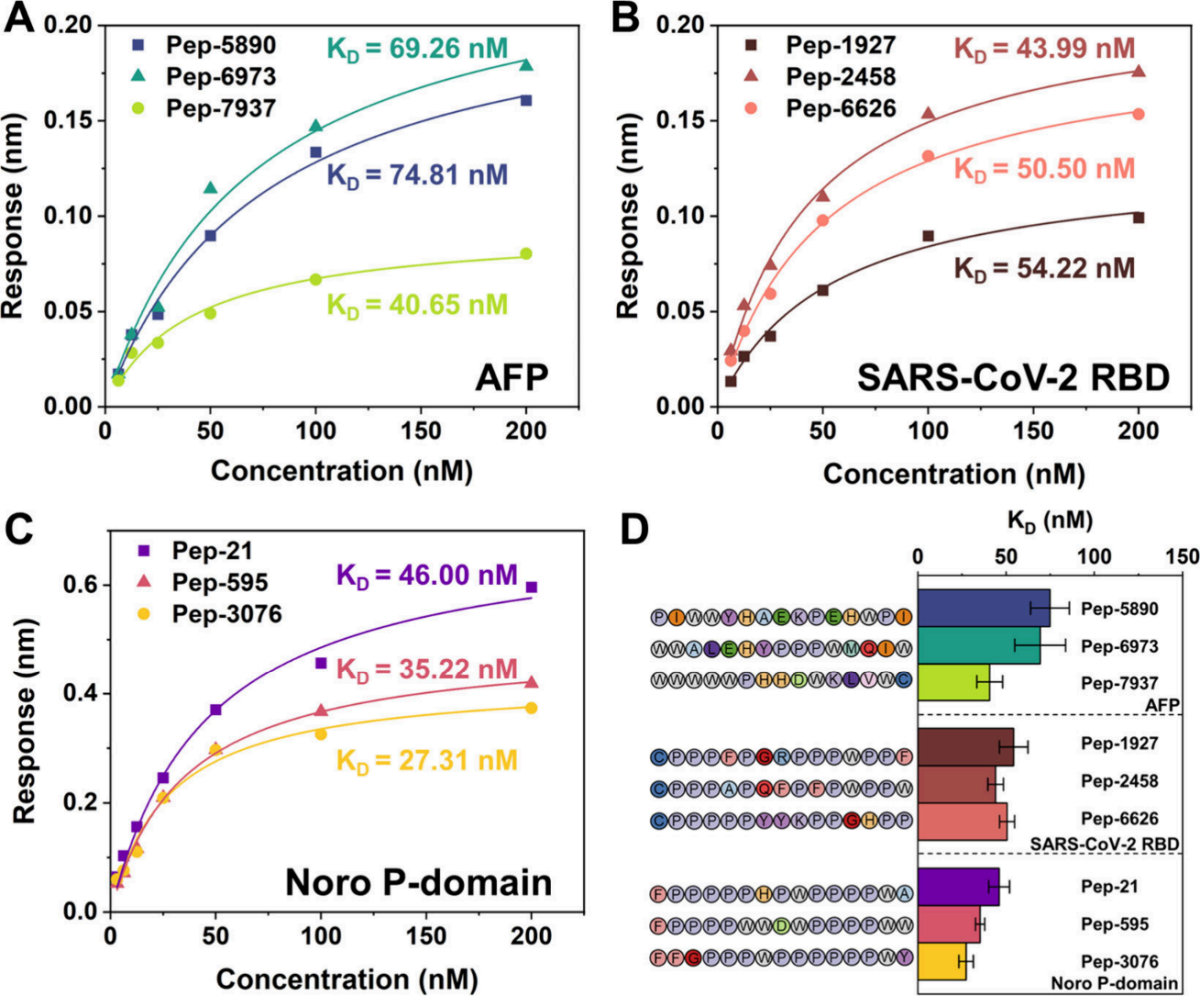
BLI characterization of the top three designs with their protein targets. (A–C) Fitting curves of the steady-state response values and the concentrations of peptides binding with (A) AFP, (B) RBD of SARS-CoV-2, and (C) Noro P-domain. (D) *K*_D_ values for the interactions between the peptides and the target proteins.

We assessed the binding affinity of the top three designs for each target protein by monitoring the changes in the BLI response. For the 15-mer peptides, both association and dissociation were completed within 60 s, hence adopting the rapid adsorption and dissociation method. For each target, three peptides, with concentrations ranging from 3.125 to 200 nM, were selected for validation, along with a random 15-mer peptide (Ran15) serving as a negative control. The negative control peptide did not exhibit affinity for any of the three targets (Figure S3). And all screened peptides demonstrated a trend where the steady-state response value increased with increasing peptide concentration, confirming their high-affinity for the targets. The steady-state response values and the affinity peptide concentrations were fitted (Figure 5A–C), and the apparent dissociation constants *K*_D_ for the binding of the peptides to their targets were determined using steady-state analysis methods. All adsorption–dissociation processed closely conformed to the binding models (Table S5, *R*^2^ > 0.98). Consequently, the *K*_D_ values for the interactions between the peptides and their target molecules were calculated to be on the order of magnitude of 10^–8^ M (Figure 5D), with Pep-3076 showing the lowest *K*_D_ (27.31 nM) interacting with the Noro P-domain.

While HTVS is faster than experimental methods, scaling it to ultralarge libraries containing millions or billions of peptides still demands significant computational resources and time, particularly when incorporating more accurate scoring functions or all-atom simulations. Using our directed mutation driven HTVS strategies, virtual peptide libraries can theoretically expand to 10^14^ members, enabling the rapid identification of peptides with the strongest binding affinities for diverse environmental and health applications. However, achieving greater structural accuracy and improving affinity predictions to fully capture the complexity of molecular interactions are essential for further enhancing the screening performance. These advancements will not only increase the reliability of HTVS in selecting optimal candidates but also drive the discovery of novel peptides with unprecedented precision and efficacy, accelerating their development for real-world applications in therapeutics, diagnostics, and environmental interventions.

## Conclusion

This study successfully demonstrated the effectiveness of directed mutation driven HTVS strategies in screening ultrahigh affinity peptides against different target proteins. Through six generations of iterative mutation and screening, we identified peptides with significantly reduced binding energy from the original random peptide library, and these peptides showed diversity in physical and chemical properties. BLI analysis further verified the high affinity between these peptides and target proteins, and the dissociation constant *K*_D_ value reached the nanomolar level. These results demonstrate the universality and scalability of our approach, providing an efficient and cost-effective way to rapidly develop novel diagnostic and treatment tools, especially in the face of sudden pandemic outbreaks, where this strategy shows the potential for rapid response.

## Supporting Information Available

Experimental details, materials, and methods, conventional serial operation mode of virtual screening, PepATTRACT affinity scores, BLI assay results, Student’s *t* test of binding energy, physical and chemical properties of peptides, parameters of BLI binding fitting models, 3D modeling method of peptide library, determination of the docking boxes, computing cluster environment configuring and HTVS method, and materials and reagents for BLI experiments (PDF)
