# Supplementary material for: High-Affinity Peptides for Target Protein Screened in Ultralarge Virtual Libraries
Source: ACS Cent Sci. 2024 Nov 2;10(11):2111–8. doi: 10.1021/acscentsci.4c01385 (PMC11613273; doi:10.1021/acscentsci.4c01385)
Supplement: Supplementary file 1 — oc4c01385_si_001.pdf [file oc4c01385_si_001.pdf]

Supporting Information

High-Affinity Peptides for Target Protein Screened in Ultralarge Virtual Libraries

Boyuan Xue,[†] Ruixue Li,[†] Zhao Cheng,[†] Xiaohong Zhou[*,†]

[†] Center for Sensor Technology of Environment and Health, School of Environment, Tsinghua University, Beijing 100084, China

[*] **E-mail:** xhzhou@mail.tsinghua.edu.cn.

Table of Contents

1. Methods S3

2. Figures S6

3. Tables S9

4. Notes S14

5. References S18

1. Methods

1.1 Construction of the Origin Peptide Library

We developed a Matlab program to automatedly generate a 10^4 random 15-mer peptide library, i.e. 10 000 possible combinations of strings containing 15 letters among the 20 one-letter code of the amino acids. The resulting 10^4 random peptides were built to three-dimensional scaffolds by using the PeptideBuilder tool on an 8-core 16-thread Think Station workstation (Windows 10 and Ubuntu 14).[1] PeptideBuilder is a Python tool for polypeptide modeling that includes all amino acid bond lengths and angles information. The large-scale peptide structure modeling based on PeptideBuilder was performed in the Ubuntu terminal, and the specific modeling method is described in **Note S1**.

1.2 Generation of Mutant Peptide Libraries

We developed a Matlab program to automatedly generate a 10^4 random mutant peptide library. The top 1% designs in the rankings (i.e. 100 sequences in total) were selected for the subsequent random mutation due to their significant binding potential. To increase the sequence diversity of the mutant peptide library, each amino acid in the 15-mer peptide had a 20% probability of being randomly replaced by another amino acid. In each round of mutation, each peptide generated 100 new mutant peptides, forming a new generation of 10^4 random mutant peptide library. We adopted an iterative evolution approach with a total of six generations of mutations, to continuously explore and expand the range of potential high-affinity peptides.

1.3 HTVS Based on a Computing Cluster

Autodock Vina 1.2.3, an open-source software allowing for docking and scoring of protein and its ligand libraries in batch mode,[2] was used to perform HTVS. Autodock Vina needed receptor (i.e. target protein) and its ligand (i.e. peptide) representations in a file format called pdbqt, which is a modified protein data bank format containing atomic charges, atom type definitions and, for targets, topological information (rotatable bonds).[3] Before docking, it was needed to add polar hydrogen atoms, assign

Gasteiger charges, merge nonpolar hydrogen atoms, and assign AutoDock Vina compatible atom types to the receptor files. These file preparations were carried out in batches using scripts from the Autodock Tools package MGLTools 1.5.7.[4] In addition, Pymol2.0.4 and the plug-in Getbox were used to clean the receptor protein and generate the location information of the docking box (**Note S2**). The virtual screening of 10^4-order peptide libraries by molecular docking technology was carried out on the Bkunyun supercomputer platform (Shenzhen, China). Specifically, Autodock Vina interconnection environment was configured in the computing cluster, scripts were submitted using SLURM commands, and CPU nodes were invoked to perform kilocore level parallel computing (**Note S3**).

1.4 High-Affinity Peptide Screening

After six generations of mutation, the top 0.3% designs were selected for further analysis (30 in total). We firstly extracted their docking conformations and amino acid sequences from the pdbqt files. The main physicochemical properties of peptides, including molecular weight (M.W.), hydrophobicity and net charge (pH 7.0), were then calculated by using R language peptides library. And the hydrophobicity was expressed by the grand average of hydropathicity (GRAVY). The pepATTRACT web server was used for peptide-protein global docking and the best conformational affinity scores were ranked to screen the candidate peptides. If there were multiple candidate peptides with similar best scores (the difference was less than 0.1), the top 10 average affinity scores were re-ranked for selection. For each target protein, the top 3 designs were finally selected for subsequent biolayer interferometry (BLI) analysis.

1.5 BLI Analysis

BLI assays were performed on an Octet Red96 instrument (ForteBio, USA) at room temperature (25°C±1°C). Target protein was biotinylated and immobilized on super-streptavidin (SSA) sensors, and peptides were used for the association and dissociation process. 1× kinetics buffer (10 mM PBS, 0.1% BSA, and 0.05% Tween-20) was used for the assays. Other Materials and reagents used in BLI experiments are shown in **Note**

S4. For each measurement, the kinetics buffer was passed for 600 s firstly to establish a stable baseline, followed by the 60-s fast association and 60-s fast dissociation. Notably, a control set of blank sensors (without immobilizing the target protein) was set to collect the data after background deduction of the non-specific adsorption signal. Data were aligned using a 1:1 binding model with Octet Analysis Studio 13.0 data analysis software. The maximum steady-state response signal (R_{max}) and equilibrium dissociation constant (K_D) can be obtained by fitting the affinity peptide concentration (Conc.) and the steady-state signal (R) of the concentration as below.

$$R=\frac{R_{max}\times \text{Conc.}}{K_D+\text{Conc.}}$$

2. Figures

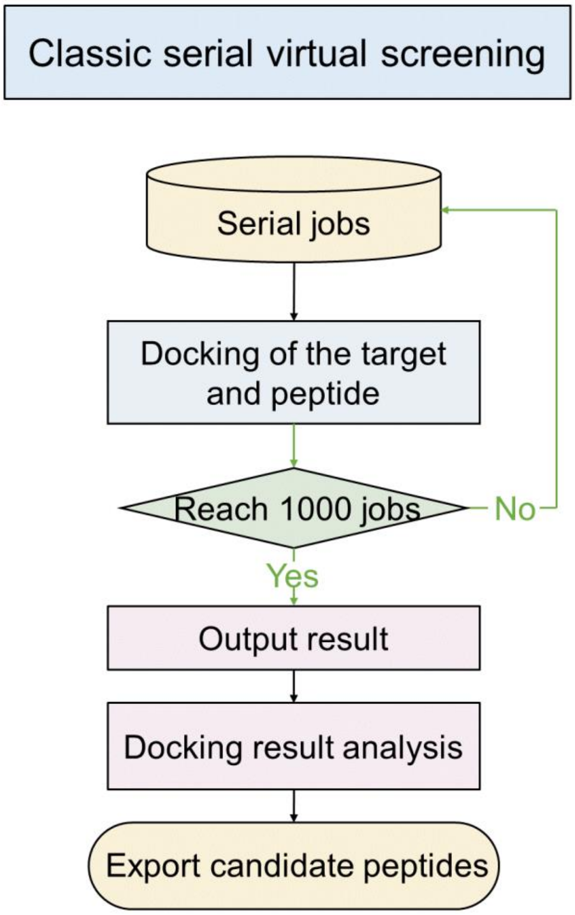

Figure S1. Conventional serial operation mode of virtual screening using Autodock Vina.

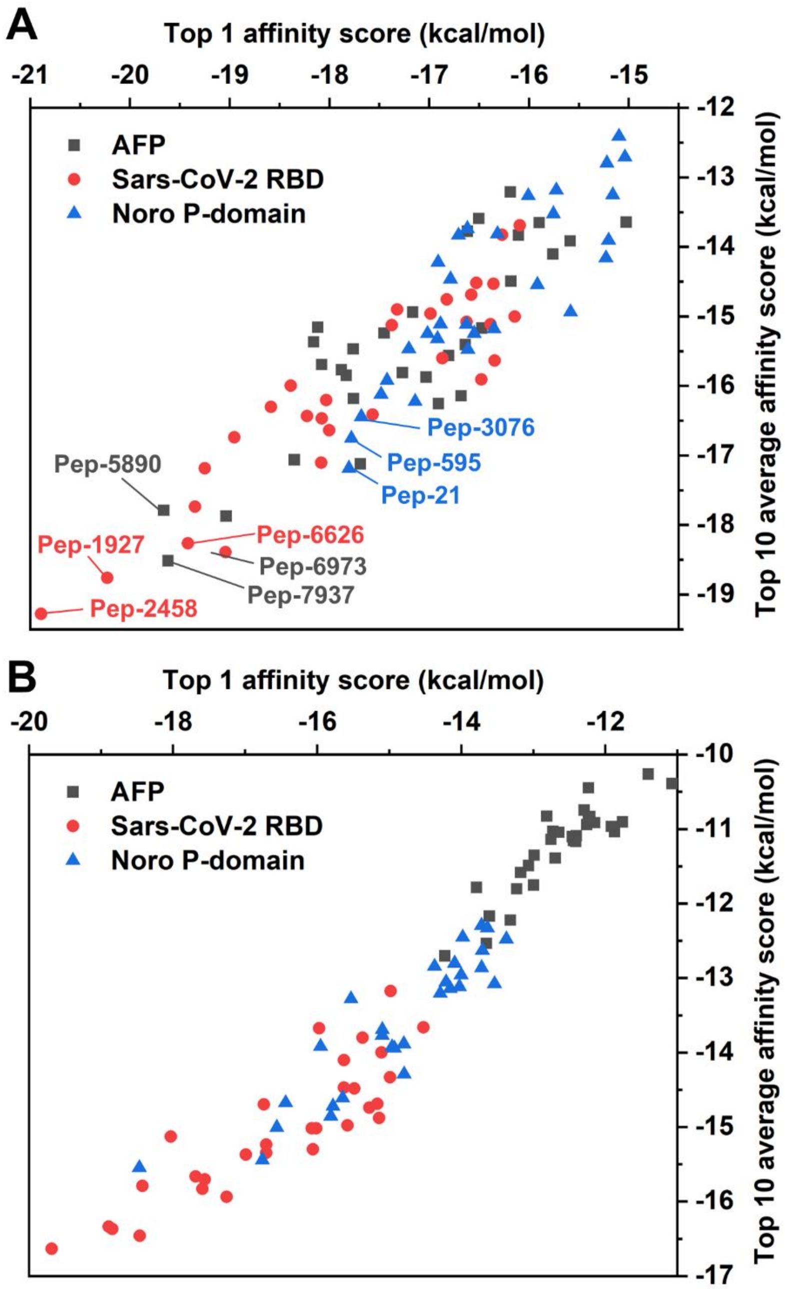

Figure S2. Top 1 and top 10 average PepATTRACT affinity scores of top 0.3% peptides of the best generation (A) and G0 (B) binding with AFP, SARS-CoV-2 RBD, and Noro P-domain.

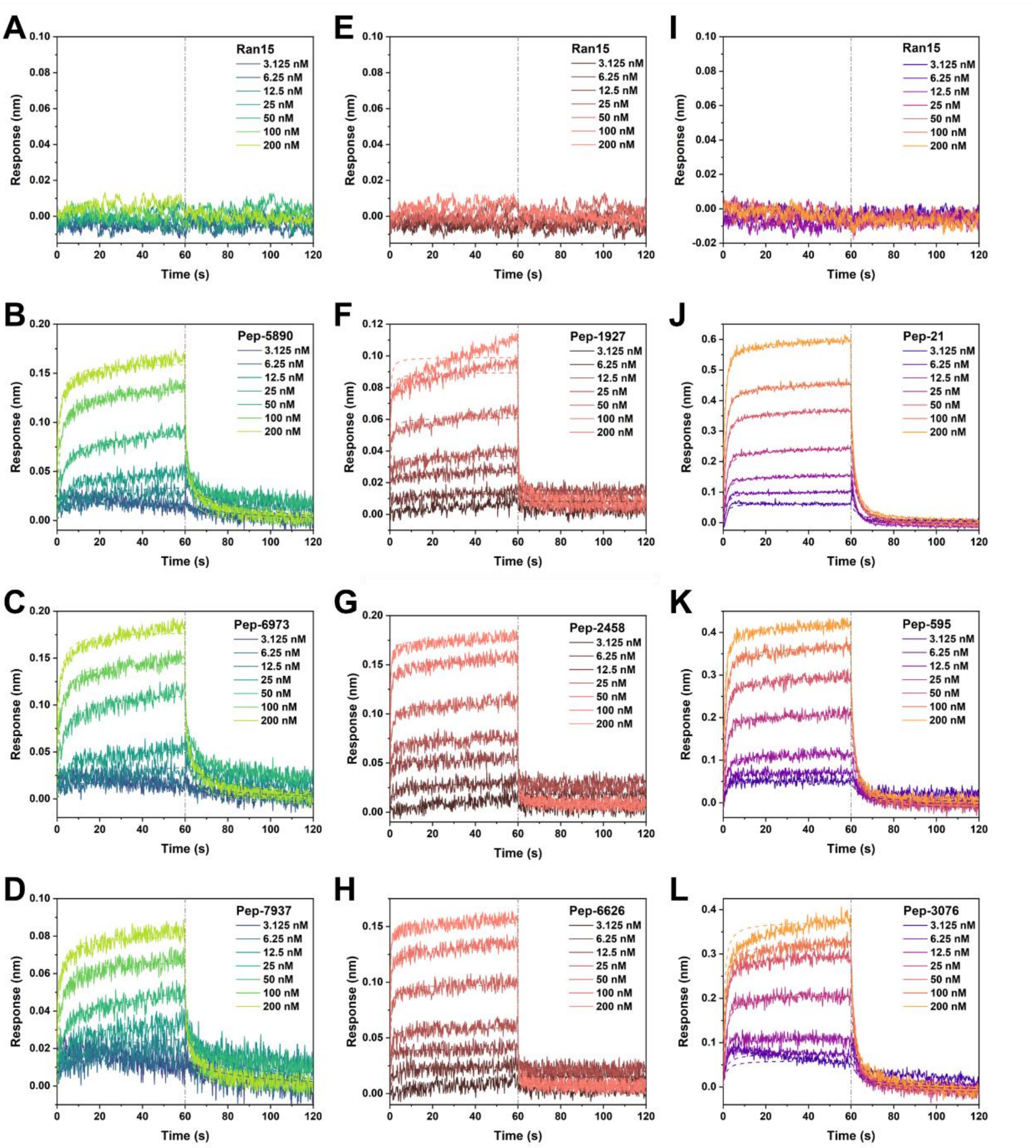

Figure S3. BLI assay results of peptides in different concentrations binding with three targets. (A-D) Ran15 (a random 15-mer peptide, A), Pep-5890 (B), Pep-6973 (C), and Pep-7937 (D) binding with AFP. (E-H) Ran15 (E), Pep-1927(F), Pep-2458(G), and Pep-6626 (H) binding with Sars-CoV-2 RBD. (I-L) Ran15 (I), Pep-21 (J), Pep-595 (K), and Pep-3076 (L) binding with Noro P-domain.

3. Tables

Table S1. Student's *t*-test of binding energy between different mutant generations. one-tailed test: whether the binding energy of the generation shows a significant decrease over its predecessor. Sample size: 10^4 per generation.

Generation	AFP			SARS-CoV-2 RBD			Noro P-domain		
	Difference	*t*	p	Difference	*t*	p	Difference	*t*	p
G1	-0.45	-33.07	8.31×10^{-234}	-0.37	-40.32	7.32×10^{-302}	-0.36	-45.30	1.25×10^{-356}
G2	-0.29	-19.35	5.71×10^{-83}	-0.28	-28.58	3.62×10^{-176}	-0.34	-38.18	5.19×10^{-289}
G3	-0.24	-15.92	5.32×10^{-57}	-0.22	-21.58	3.86×10^{-102}	-0.26	-27.24	1.75×10^{-160}
G4	-0.21	-14.33	1.31×10^{-46}	-0.17	-15.52	5.53×10^{-54}	-0.32	-30.48	1.69×10^{-199}
G5	0.47	30.05	1	-0.35	-31.00	4.35×10^{-206}	-0.31	-28.15	4.73×10^{-171}
G6	-0.13	-8.31	5.13×10^{-17}	-0.05	-4.87	1.13×10^{-6}	-0.14	-12.20	4.21×10^{-34}
G6[a]	0.34	21.56	1						

[a] Compared to G4.

Table S2. Sequences and main physical and chemical properties of top 0.3% peptides of the best generation binding with AFP.

**No.**	**Sequence**	**M.W.**	**Hydrophobicity**	**Net charge (pH 7)**	**Docking score (kJ/mol)**
7937	WWWWWPHHDWKLVWC	1970	0.50	-2.05	-11.8
1775	NWFPCFWAIDDWWIA	1927	0.32	-2.05	-11.5
474	AWYDGYFGPDCWWNF	1989	0.18	-0.54	-11.5
5890	PIWWYHAEKPEHWPI	1912	0.23	1.22	-11.5
4687	GWFRGQPYFGHFWLN	1983	0.39	-0.02	-11.5
3816	WLIFDTPGPFYWRWV	2017	0.33	-0.78	-11.4
6973	WWALEHYPPPYMQIW	1974	0.11	1.45	-11.4
5856	MSWWYHAGKPQHWPY	1917	0.39	-1.08	-11.4
1751	NNFPCFWCGDWWGWV	1909	0.34	1.45	-11.1
6002	PIFWVHAYIPRHLPY	2249	0.33	0.22	-11
1522	WWWWWPYHDWKLVYP	1936	0.52	0.18	-11
1614	NWFPCHWAYLWVGWA	1834	0.30	-2.05	-10.9
1627	MQFDCFNAGDWWGWA	2180	0.73	0.18	-10.9
7707	VVLWCFWHYFWWLYM	2017	0.60	-0.09	-10.9
281	FWYIPWWVPYACWPC	1961	0.12	-1.54	-10.9
1103	YGYSGEPQWVHWWEH	1973	0.39	-2.05	-10.8
1763	NWFPCFWAGDWWGWD	1877	0.34	-0.81	-10.8
4070	VFALQQVWPEYAHCW	1907	0.28	-0.05	-10.8
5902	PIWWYCAEKPSPWPF	1843	-0.07	2.22	-10.8
5930	KIKWMPAEKPSHMPY	2016	0.01	0.46	-10.8
6283	GWALEYSWPWYQHRH	2090	0.17	-2.02	-10.7
1074	YWYEGRPGWDIWWEF	1840	0.25	-3.05	-10.7
1745	NWEPCFWAGDDWGWA	1995	0.32	-0.54	-10.7
4606	YTFDGAVYWHHWWLN	1941	0.12	-0.06	-10.7
5064	NFYYRGPYCGTWWDL	1875	0.14	1.94	-10.6
5374	TFGIKSRWACPPWYY	1850	-0.14	-1.02	-10.6
5650	YGNGWDYKADFDRGW	1986	-0.04	0.21	-10.6
601	YWYQGYPGPDHWRNF	1871	0.39	-2.05	-10.6
1764	NWGECFWAGDWWGWA	2020	0.29	1.18	-10.6
1837	YAYNKVPYWGHWWFC	1808	0.31	-2.02	-10.6

Table S3. Sequences and main physical and chemical properties of top 0.5% peptides of the best generation binding with SARS-CoV-2 RBD.

No.	Sequence	M.W.	Hydrophobicity	Net charge (pH 7)	Docking score (kJ/mol)
1298	YPPPAPYSPPPWIPQ	1764	0.33	-0.05	-9.1
2458	CPPPAPQFPFPWPPW	1741	0.44	-0.05	-9.1
3286	WPPPCPIFPPPLPPW	1689	0.17	0.95	-9.1
1927	CPPPFPGRPPPWPPF	1849	0.22	0.46	-9
9913	WPPPPPYFPPPWHHP	1762	0.01	-0.05	-9
6985	CPRPPPYDPPPWGPW	1835	0.38	-0.09	-9
9811	WFPPPPYFPPCWTCP	1885	0.18	0.42	-9
393	CPYPNPAHWPPWHPW	1644	0.03	1.18	-9
6626	CPPPPPYYKPPGHPP	1944	0.19	0.98	-9
7068	PPPPWTWFPPPWRPW	1760	0.43	-0.05	-9
1663	WFPPAPNFPFPWGPC	1687	0.32	-0.06	-8.9
1831	CPPPWPYGPPPWPPV	1758	0.33	-0.06	-8.9
2613	CFPPIPYPYPPPSPW	1804	0.15	-0.78	-8.9
432	YPPPPPYFPPPWHPD	1791	0.23	0.18	-8.9
5059	CPPPHPYPYPPWGPW	1817	0.05	0.98	-8.9
5379	WPPPAPQPYPPMRPW	1626	0.29	-1.05	-8.8
9529	CPAPAPYFPPPWEPG	1858	0.14	-0.78	-8.8
9841	WPPPPPYFPNPWHEP	1831	0.33	0.18	-8.8
9875	WPPPLPYFPPPWHCP	1673	0.24	0.91	-8.8
3254	WPPPCPIFPPPKPPC	1831	0.10	0.98	-8.8
5259	WPPPAPSPYPPWRPW	2113	0.52	0.22	-8.7
6048	WPHTPWWFPPWWGWF	1868	0.16	0.97	-8.7
7081	PPPPAYYFPPPWRPW	1702	0.32	-0.05	-8.7
1872	CPPPWPFGPPPWPQV	1643	0.40	-0.02	-8.7
2051	WPPPGAYIPPPWPPA	1861	0.33	0.97	-8.7
4749	IPPPLPYFFRPWAPY	1809	0.16	-0.82	-8.7
5044	CPPPHPYPYPDWGPW	1782	0.35	0.18	-8.7
5176	CFPPHPYPYPPWIPA	1730	0.20	0.42	-8.7
5737	HPPSAPYFPPPWHPC	2032	0.39	-0.03	-8.7
5822	WYYTPWWYPSPWGWG	1821	0.34	0.22	-8.7

Table S4. Sequences and main physical and chemical properties of top 0.5% peptides of the best generation binding with Noro P-domain.

No.	Sequence	M.W.	Hydrophobicity	Net charge (pH 7)	Docking score (kJ/mol)
1107	WPAPYAPPVPPPPWY	1717	0.28	0.22	-9.8
21	FPPPPPHPWPPPPWA	1779	0.39	-0.02	-9.5
3076	FFGPPPWPPPPPPWY	1744	0.40	-0.02	-9.5
2500	WPGPPWGPIPPPPWY	1887	0.44	-0.03	-9.4
5238	FPYPPWVPPFPPWPY	2029	0.39	-1.02	-9.4
4305	WDWPPWVPWPPPPWW	1988	0.35	-1.02	-9.3
595	FPPPPWWDWPPPPWW	1810	0.33	-0.02	-9.3
7725	WPYPPWVPPPPPWPP	1768	0.44	-0.02	-9.3
1368	FPGPWWPPIPPPPWP	1745	0.31	0.22	-9.2
29	FPPPPPHPWPPPPWV	1736	0.30	0.22	-9.2
4436	FPPPPYHPPPPPPWI	1769	0.42	0.22	-9.2
54	FPPFVPHPWPPPPWA	1664	0.27	0.12	-9.2
7113	YPPAPHVPCCPPCWP	1836	0.28	0.22	-9.2
9754	FPPPPWHPWPPPPWT	1785	0.45	-0.02	-9.2
118	FPGIPWPPPSPPFWY	1759	0.27	-0.02	-9.1
167	FPPPPWPPPSPPPWY	1744	0.40	-0.02	-9.1
2454	WPGPPWGPIPPPPWY	1901	0.37	-1.02	-9.1
594	FPPPPWVDWPPPPWW	1792	0.13	0.98	-9.1
6532	FPGPPWTPPRPPPWY	1736	0.14	0.98	-9.1
69	FPPPPPRPWPPPPWA	1688	0.49	-0.02	-9.1
1381	FPGPWWGPIPPPPWG	1906	0.47	-0.06	-9
1700	FYGWCWPPPIPPPWY	1838	0.41	-0.02	-9
1710	FLGPPWPPWPTPPWY	1783	0.38	-0.02	-9
1757	FPGPPWPPFPTPPWY	1834	0.45	-0.02	-9
384	FPGPPWWPIPPPPWY	1617	0.22	-0.02	-9
3856	PPGPPWPPTPPPPWP	1717	0.28	0.22	-9
45	FPPPPPHPWPPPPWA	1736	0.30	-1.02	-9
717	WPGPPWPPIPPPPWD	1784	0.38	-0.02	-9
719	WPGPPWPPIPPPPWY	1751	0.23	-1.02	-9
8564	GPYPYWPPAPPPEWP	1792	0.32	0.22	-9

Table S5. R_{max} K_D, and R^2 of BLI binding fitting models.

**Target**	**Peptide**	**Sequence**	**R_{max} (nm)**	**K_D (nM)**	**R^2**
AFP	Pep-5890	PIWWYHAEKPEHWPI	0.2242±0.0144	74.81±11.04	0.9979
	Pep-6973	WWALEHYPPPYMQIW	0.2449±0.0216	69.26±14.38	0.9928
	Pep-7937	WWWWWPHHDWKLVWC	0.0945±0.0061	40.65±7.31	0.9903
SARS-CoV-2 RBD	Pep-1927	CPPPFPGRPPPWPPF	0.1296±0.0076	54.22±8.13	0.9855
	Pep-2458	CPPPAPQFPFPWPPW	0.2149±0.0080	43.99±4.43	0.9852
	Pep-6626	CPPPPPYYKPPGHPP	0.1940±0.0063	50.50±4.29	0.9884
Noro P-domain	Pep-21	FPPPPPHPWPPPPWA	0.7092±0.0344	46.00±5.92	0.9931
	Pep-595	FPPPPWWDWPPPPWW	0.4957±0.0130	35.22±2.65	0.9957
	Pep-3076	FFGPPPWPPPPPPWY	0.4267±0.0208	27.31±4.09	0.9942

4. Notes

Note S1. Specific 3D modeling method of peptide library.

After installing the PeptideBuilder tool in Python 3.8, the specific 3D modeling method is as follows:

(1) Call the PeptideBuilderku and Geometry libraries.

(2) Read the peptide sequence line by line from the peptide library file using the for loop and utf-8 character set, and store the sequence of each row as a string in the AA_chain.

(3) Use Geometry.geometry function to call the initial geometry information of amino acids according to the first positions of AA_chain, and use PeptideBuilder. Initialize_res function to generate the corresponding 3D structures.

(4) Use PeptideBuilder.make_extended_structure function to read the subsequent amino acid sequences and generate the corresponding 3D structures of the complete peptides.

(5) Call the Bio.PDB library, use the Bio.PDB.PDBIO function to generate output results and finally save them into a pdb file.

Note S2. Determination of the docking boxes.

In the process of structure preparation, the receptor proteins for virtual screening were obtained from the crystal structures, the protein structures were cleaned by the cleaning option of Pymol 2.0.4. The water molecules and small molecular ligands in the structures were manually checked to remove, and the generated structure were saved in the form of pdb file.

The directed mutation driven HTVS generated docking boxes centered on receptor proteins. The Getbox plugin of Pymol 2.0.4 was used to automatically form the docking box parameters. The information of the boxes is as follows:

Target	Docking box parameter	Coordinate value (x, y, z)
AFP	Center	(81.2, 72.9, 76.3)
	Size	(67.5, 88.9, 102.6)
	Range	(47.45−114.95, 28.45−117.35, 25−127.6)
SARS-CoV-2 RBD	Center	(179.5, 110.7, 260.2)
	Size	(56.0, 54.0, 68.6)
	Range	(151.5−207.4, 83.7−137.7, 225.9−294.5)
Noro P-domain	Center	(54.1, 54.6, 40.9)
	Size	(41.6, 33.1, 44.2)
	Range	(24.05−84.15, 25.2−84, 13.25−68.55)

Note S3. Computing cluster environment configuring and HTVS method.

In terms of the computing cluster environment configuring, multiple CPU nodes were invoked in the Bkunyun general computing area, and every node was configured as 80 core, single-core 2G memory, and 3.5GHz. The number of called nodes was set according to the scale of simultaneous computation, and one 80-core node was called for every 2500 peptide-protein docking processes, with 80 processes executed in parallel. All interconnection tasks and node invocations are managed in the Simple Linux Utility for Resource Management (SLURM) system. The interconnection processes were executed by shell scripts, with the srun command to realize parallel running and the sbatch command to submit to the computing cluster.

Virtual screening of peptide libraries was performed using Autodock Vina. The docking receptor option was set to the processed receptor protein pdbqt file, the docking ligand option was set to the processed peptide pdbqt file, the docking box option was set according to the coordinates in **Note S2**, and the exhaustiveness parameter was set to 1. All other parameters were set to default, the peptide library was traversed through the loop command.

Note S4. Materials and reagents for BLI experiments.

Alpha fetoprotein, SARS-CoV-2 RBD protein and Norovirus GII.4 VP1 P-domain protein were purchased from Sino Biological Co., LTD. (Beijing). All peptides were purchased from GenScript Biotechnology Co., LTD. (Nanjing) and were chemically synthesized by solid-phase synthesis. 10 mM PBS buffer (10 mM sodium phosphate, 25 mM sodium chloride, pH 7.2) was prepared using BupH buffer powder packs from Thermo Fisher Technology Co., LTD. (Shanghai). 1 x kinetic buffer (10 mM PBS, 0.1% BSA and 0.05% Tween-20) was laboratory made. Biotinizing reagent EZ-Link NHS-biotin was purchased from Thermo Fisher Technology Co., LTD. (Shanghai). Other chemicals were obtained from Sigma Aldrich Co., LTD. (Shanghai), and all solutions were prepared using molecular bio-grade ultra-pure water (Corning, Shanghai). Prior to experiments, reserve solutions of target proteins (0.25 mg/mL) and peptides (1 mg/mL) were prepared in ultrapure water and stored at -80 °C until diluted to the desired concentration with kinetic buffer solution.

5. References

1. Tien, M. Z.; Sydykova, D. K.; Meyer, A. G.; Wilke, C. O., PeptideBuilder: A simple Python library to generate model peptides. *PeerJ* **2013,** *1*, e80.

2. Eberhardt, J.; Santos-Martins, D.; Tillack, A. F.; Forli, S., AutoDock Vina 1.2.0: New Docking Methods, Expanded Force Field, and Python Bindings. *J. Chem. Inf. Model.* **2021,** *61* (8), 3891-3898.

3. Seeliger, D.; de Groot, B. L., Ligand docking and binding site analysis with PyMOL and Autodock/Vina. *J. Comput.-Aided Mol. Des.* **2010,** *24* (5), 417-422.

4. Morris, G. M.; Huey, R.; Lindstrom, W.; Sanner, M. F.; Belew, R. K.; Goodsell, D. S.; Olson, A. J., AutoDock4 and AutoDockTools4: Automated docking with selective receptor flexibility. *J. Comput. Chem.* **2009,** *30* (16), 2785-91.
